# Reflections on physical activity intervention research in young people – dos, don’ts, and critical thoughts

**DOI:** 10.1186/s12966-016-0348-z

**Published:** 2016-02-18

**Authors:** Esther M. F. van Sluijs, Susi Kriemler

**Affiliations:** MRC Epidemiology Unit & UKCRC Centre for Diet and Activity Research (CEDAR), University of Cambridge School of Clinical Medicine, Box 285, Addenbrookes Hospital, Hills Road, Cambridge, CB2 0QQ UK; Epidemiology, Biostatistics and Prevention Institute, University of Zürich, Hirschengraben 84, CH-8001 Zürich, Switzerland

**Keywords:** Physical activity, Children, Adolescents, Intervention, Behaviour, Methodology

## Abstract

**Background:**

Physical activity has been associated with many benefits throughout the life course. As levels of physical activity appear to be insufficient in large populations, the development of effective interventions to promote or maintain activity levels in young people are therefore of key public health concern. Physical activity intervention research in young people is challenging, but this should not be a reason to continue conducting inferior quality evaluations. This paper highlights some of the key issues that require more careful and consistent consideration to enable future research to achieve meaningful impact.

**Discussion:**

This paper critically evaluates, amongst others, current research practice regarding intervention development, targeting, active involvement of the target population, challenge of recruitment and retention, measurement and evaluation protocols, long-term follow-up, economic evaluation, process evaluation, and publication. It argues that funders and researchers should collaborate to ensure high quality long-term evaluations are prioritised and that a trial’s success should be defined by its quality, not its achieved effect.

**Summary:**

The conduct and publication of well-designed evaluations of well-defined interventions is crucial to advance the field of youth physical activity promotion and make us better understand which intervention strategies may or may not work, why, and for whom.

## Background

Physical activity has been associated with many benefits throughout the life course. In young people, this includes improved mental health [1], motor skill development [2], cardiovascular risk profile [3], weight development [4], bone health [5], cognitive functioning [6], and quality of life or self-esteem [7]. However, levels of physical activity appear to be insufficient in large populations [8] and decline rapidly from late childhood through adolescence [9]. The development of effective interventions to promote or maintain activity levels in young people are therefore of key public health concern [10, 11].

The past 20 years has seen an explosion of publications on physical activity interventions in young people. There is also a concomitant increase in the reviews of these evaluations, many of which identify methodological weaknesses related to both internal and external validity [12–15]. Despite these frequent calls for improved methodological rigour to enable establishment of a higher quality evidence base, many studies continue to add to the existing mountain of poor evidence. This leaves the evidence base fragile and open to substantial criticism and debate [12, 13, 16, 17], and provides challenges to truly inform policy and practice on what action to take. Based on our own experience of reviewing the evidence and (the challenges of) conducting intervention research in young people, this commentary aims to provide a critical platform for those embarking on or engaged in intervention research, and argues why and how rigour throughout the intervention research process matters. This includes intervention development and implementation, evaluation and dissemination. The issues described below may seem obvious for many, and are certainly not new. But for a variety of reasons they are too often ignored or flawed, which may ruin study conduct, result in false negative or positive findings, and provide moot new knowledge.

## Discussion

### Investing in the future

#### To map or not to map?

The use of structured processes to guide intervention development is becoming increasingly more common, which we welcome [18, 19]. When thoughtfully considered and well-conducted, it provides a useful guide to developing an intervention based on the best available and most relevant evidence. However, they are at risk of becoming a ‘completion’ exercise (exemplified by repetitive performance objectives and matrices), suggesting a premise of rigorous development but failing to deliver. Yet, when conducted well, it helps ensure the intervention targets the most important and salient behaviours and is acceptable to those delivering and experiencing it, creates a pathway to understanding how the intervention may work (by developing a hypothesised causal model), and helps identify gaps to be addressed prior to embarking on full scale evaluations.

#### The whole forest or just a tree?

As public health gains are thought to be greatest when achieving population-level shifts in exposures with minimal harm [20], targeting whole populations may be our best bet. In contrast, certain population subgroups may benefit most from increased physical activity, such as girls and overweight youth, and targeting them may help reduce health inequalities. Review evidence suggests that girls particularly benefit from single-sex interventions [21, 22], although recent primary research indicates that both sexes benefit from population approaches, with girls benefitting more than boys [23, 24]. Although targeted interventions may be ‘easier’ to implement than population-based approaches, they also tend to be more intensive for the participants to engage with (e.g. face-to-face session vs. environmental change). The lack of like-for-like comparisons makes it difficult to draw firm conclusions regarding the need for, and value of, targeting. In addition, research into other behaviours suggests that a whole population approach overcomes isolation, stigmatization and detrimental mental health consequences [25], an issue particularly relevant in adolescent and overweight populations but largely ignored in the physical activity literature. Due consideration should therefore be given to the need for a universal or targeted approach, the acceptability and effectiveness of different approaches available to targeting, and the potential positive and negative consequences of either.

#### Listen to others and their opinions

As researchers we make decisions about other peoples’ lives, expecting them to be keen on adopting our ideas. It is therefore crucial that from early in the research process we seek and integrate the views of those expected to deliver and participate in the intervention, both through research (e.g. focus groups) and in an advisory role (e.g. Patient and Public Involvement). Qualitative research is increasingly included in intervention projects, but there are few examples where early and ongoing engagement has substantially influenced the predefined intervention or evaluation [26]. Active engagement however aids the development of an acceptable and attractive intervention (and evaluation) strategy, and increases the likelihood of a successful trial, effectiveness, and intervention sustainability. Well-conducted feasibility and pilot trials with detailed process evaluations are therefore a crucial part of the trial development process [18, 27].

#### Fun, fun, fun

If asked, the magic intervention ingredient identified by young people is FUN [28, 29], whether it is with their family or with friends. The big step-up for intervention research is to find a focus resulting in sustained engagement rather than just momentary fun. Various psychological models link sustained enjoyment to autonomous forms of motivation (e.g. Self Determination Theory) [30], also in young people [31]. Interventions targeting autonomous forms of motivation, such as identified regulation and intrinsic motivation, have shown promise, particularly in changing specific physical activity behaviours (for example in the context of physical education) and for adolescents [31]. For promoting overall physical activity, the magical task therefore lies in finding the ideal (combination of) interventions to foster this fundamental psychological need. This may range from curriculum changes (controlled motivation with wide reach) to providing free choice (volitional motivation with potentially limited reach). Whatever it will be, an intervention delivering on sustained fun is likely to be engaging and to promote ongoing involvement, while being enjoyable to deliver and evaluate.

### Going the extra mile

A variety of logistical and resource constraints (e.g. availability of objective monitors, financial limitations, staff availability, potential for blinding), time limitations (e.g. ethical approval, recruitment challenges, school term restrictions, time-limited funding), and ethical considerations (e.g. consent requirements, measurement burden) often underlie compromises in methodological quality. Although understandable, continuing on this trajectory will unlikely lead to significant advancements. Substantial investment in fewer well-prepared and feasibility-tested high quality evaluations is therefore needed. Whether an efficacy trial or pragmatic evaluation, set in school or community settings, these projects should focus on evaluating well-defined interventions in representative samples, including valid and preferably objective measures along the hypothesised causal pathway and ensuring high internal and external validity.

#### To recruit and to retain

Recruitment and retention is challenging, particularly when working with young people. We face a challenge of changing attitudes; from overly enthusiastic children to the overarching adolescent tendency of denial. Substantial investment in optimising participant recruitment and retention is worthwhile, while at the same time being realistic about what is achievable (to funders) and ensuring continued participation across all population subgroups to optimise external validity. Surprisingly little is known about effective recruitment and retention [32], and an increased understanding of effective ways of retaining adolescent participants over time is urgently needed. We therefore strongly encourage researchers to accurately report on their recruitment and retention strategies and their actual or perceived effectiveness to inform future trials. It is important to specifically consider retention on the main outcome measure, particularly where this involves objectively measured physical activity. Issues with monitor refusal, non-return, non-wear or insufficient wear time rapidly reduces the sample available for the main effect analysis, and the study’s ability to draw valid conclusions.

#### Timing is everything

Many projects depend on the school year, even when the school is not used as the setting for intervention delivery, limiting study time to around 35 ‘normal’ weeks. As project delays are not uncommon (delayed ethical approval, challenging recruitment, or intervention implementation issues), this time limitation encourages some to prioritise collecting baseline data in intervention participants. Although understandable, this imbalance can compromise data comparability and study power. Diverse weather conditions, differential monitor reactivity, and other disturbances, such as ‘special days’ held at typical times of the year (e.g. parties and sports days), can result in unwanted group differences. Simultaneous data collection in all study groups and scheduling multiple measurement periods within individual schools [33] helps to minimize the impact of these disturbances, and should be considered.

#### Keep it real

While there is utility in showing that an active travel intervention increases frequency of active travel, whether it actually impacts on total daily physical activity should be assessed as well. Behavioural compensation at other times of the day has been observed, and may partially explain the limited effect observed when physical activity is measured at day-level [16]. An additional investigation of the impact on more upstream ‘harder’ outcomes (e.g. cardiovascular indicators, academic/cognitive performance, aerobic fitness), well-being, and time spent sedentary will create a fuller picture of how our interventions impact children’s lives and health, and may help generate traction at the policy level.

#### Happily ever after…?

Assessing the longer-term impact of youth physical activity interventions is widely considered important [11, 12], but rarely done [34]. Anecdotal evidence highlights challenges with obtaining funding, participant retention, and publication (due to retention issues and null-findings) as reasons for this lack of evidence. Planning for long-term follow-up at the design and funding stage is one important way of overcoming barriers. Importantly, funding agencies should consider setting aside funds for the long-term follow-up of high quality informative trials, or supporting fewer trials in total but more with long-term measures. Although some argue that long-term follow-up is most relevant when studies show short-term effects [34], we argue that follow-up should be contingent on the methodological quality of the original trial, irrespective of effect. For example, sufficient time needs to have lapsed to be able to observe health effects based on subtle behavioural changes that may even have a lag-time to effects themselves [35].

#### Money makes the world go round

To truly influence policy and practice, it is insufficient to solely continue focussing on physical activity effects. Inclusion of health-related outcomes and an economic evaluation, particularly where the intervention involves multiple agencies and delivers, is essential [14]. The few cost-effectiveness evaluations available indicate that physical activity promotion in young people is likely to be cost-effective [14, 36]. Building up a solid evidence base based on high quality evaluations is likely to create substantial political traction and will guide decisions on utilisation of limited public health expenditure [37, 38].

### Never-ending story?

Studying the effect of the intervention on physical activity behaviour and key secondary outcomes (e.g. health markers) is a logical priority in the evaluation process. However, in order to truly progress our understanding of how and why interventions do (or do not) work and how they might need to be adapted, continued evaluation is crucial.

#### The intervention delivery intention-behaviour gap

The extent to which intervention components were delivered in the manner intended, is poorly understood and rarely documented [39]. Process evaluations aid examination of the quantity and quality of what was actually implemented in practice, and why [40, 41]. Despite best intentions, differing contexts such as practical challenges and personal/institutional preferences commonly lead to varied delivery across settings. Understanding this variation, its reasons and consequences using mixed methods research in the context of a high quality evaluation will improve interpretation of intervention effects, and the development and delivery of future interventions. Improved understanding of the importance of context for intervention implementation and effect will also help explain why replication trials commonly do not replicate the original trial findings. In the school setting, the role of professional development and motivation of the teachers responsible for intervention delivery is particularly crucial. We additionally need to further develop our thinking on how interventions can be developed more flexibly to allow for adaption to context, and with that possible higher acceptability, and the implications this has for evaluation designs.

### *Look under the bonnet*

Our understanding of what intervention elements are effective, and how, is limited at best [42, 43]. Designing a study to enable the investigation of intervention mediation is becoming more common, but is still not commonplace. A key prerequisite is the inclusion of measures along the hypothesised causal pathway, included at appropriate times and in all study populations. This should include measures of potential mediating mechanisms, the specific behaviours targeted, objectively measured *total* physical activity, and objective measures of more distal outcomes. Pre-specified and adequately powered analyses plans should not only focus on the main intervention effects, but also on studying the mediating mechanisms and potential moderators, or subgroup effects [44]. Only then can we start to answer the question: “For whom, why, and under what circumstances do interventions work?”

### *The whole truth, and nothing but the truth*

Finally, treat your data with the respect it deserves. Plan for your analyses before seeing the data, and stick with it. Be clear and consistent about primary and secondary outcomes, and pre-plan subgroup analyses. An intervention project is a rich resource that can give rise to a large number of publications focussing on a diverse set of research questions. Despite the demand of high-impact journals towards minimalistic paper length, irrespective of the outcome we recommend publishing a single paper with the main findings for all primary and secondary outcomes, including potential moderating effects [45]. Only by applying consistent and rigorous processes to the statistical analyses and subsequent unrestricted reporting of the results, whatever they are, can we truly understand the extent and complexity of the intervention effects.

## Conclusions

In this paper, we have outlined some of the key areas we believe the research field of physical activity promotion in young people may want to focus their efforts on in order to increase the quality and potential impact of the work produced. Table 1 provides a summary of the actions that should be considered to improve the level of evidence produced by future research. Although there are other issues to be addressed and alternative actions in relation to the key issues discussed are entirely plausible, taking the actions suggested will lead to a major step forwards in increasing the quality of the current evidence base.

**Table 1:** Suggested actions for improving the level evidence for physical activity interventions in young people (structured by intervention mapping (IM) steps)

IM step	Suggested actions to be taken	Paragraph link
Needs assessment	Ensure timely input from your stakeholders at key points in the research process. Public Involvement, qualitative research and process evaluations will help inform and adapt interventions *and* evaluations to make them acceptable and relevant to the target audience and feasible for implementation, but only if you take into account the information it produces.	Listen to others and their opinions
Matrices	Carefully consider your target population and the potential negative and positive consequences of targeting. Consider comparing the feasibility, acceptability, and effectiveness of targeted vs. non-targeted interventions in a trial.	The whole forest of just a tree?
Theory	Use relevant theory, and feedback from your target audience, to understand how we might be able to achieve sustained engagement through, for example, focusing on autonomous forms of motivation.	Fun, fun, fun
Program	Carefully design your intervention and link it to a pre-defined causal model. This helps guide your evaluation and improve our understanding of causation.	To map or not to map?
Implementation	Plan ahead for the inclusion of a detailed (mixed-methods) process evaluation to allow for a deeper understanding of the (lack of) intervention effect.	The intervention delivery intention-behaviour gap
Evaluation	Implement recruitment and retention strategies based on the best available evidence, and report on them and their effectiveness to inform future research.	To recruit and to retain
	Consider how to schedule measurements to avoid bias due to for example weather or influences of ‘special days’.	Timing is everything
	Include upstream outcomes, preferably objectively-measured – this is likely to increase impact at the policy and practice level.	Keep it real
	Don’t miss the often “neglected” assessment at long-term follow-up - make early provisions.	Happily ever after…?
	Include evidence for policy makers that the intervention is worth it. This may involve collaboration with health economists to evaluate the cost-effectiveness of your intervention.	Money makes the world go round
	Design your evaluation to enable studying mediators and moderators. Use your pre-defined causal model and consider the timing of assessment and statistical power.	Look under the bonnet
Dissemination	Report everything, preferably in a single paper, and tell it as it is to increase our collective understanding of the complexity of intervention effects.	The whole truth, and nothing but the truth

We argue that researchers and funders should prioritise the conduct and publication of high quality and long-term evaluations of physical activity promotion in young people, while reducing the output of lower quality ones. In this situation, whether or not the trial is successful should be defined by the delivery of a well-defined intervention in the context of a high quality evaluation, including a process evaluation and use of validated measures of factors along the hypothesised causal pathway. Irrespective of effect, the conduct and publication of well-designed evaluations of well-defined interventions is crucial to advance our field and make us better understand which intervention strategies may or may not work, why, and for whom.
